# Weight Gain During Adulthood and Body Weight at Age 20 Are Associated With the Risk of Endometrial Cancer in Japanese Women

**DOI:** 10.2188/jea.JE20110020

**Published:** 2011-11-05

**Authors:** Satoyo Hosono, Keitaro Matsuo, Kaoru Hirose, Hidemi Ito, Takeshi Suzuki, Takakazu Kawase, Miki Watanabe, Toru Nakanishi, Kazuo Tajima, Hideo Tanaka

**Affiliations:** 1 Division of Epidemiology and Prevention, Aichi Cancer Center Research Institute, Nagoya, Japan; 2 Department of Epidemiology, Nagoya University Graduate School of Medicine, Nagoya, Japan; 3 Department of Planning and Information, Aichi Prefectural Institute of Public Health, Nagoya, Japan; 4 Department of Medical Oncology and Immunology, Nagoya City University, Nagoya, Japan; 5 Department of Gynecologic Oncology, Aichi Cancer Center Hospital, Nagoya, Japan; 6 Director, Aichi Cancer Center Research Institute, Nagoya, Japan

**Keywords:** endometrial cancer, weight gain, case-control study

## Abstract

**Background:**

Current obesity is an established risk factor for endometrial cancer; however, the roles of weight gain during adulthood and obesity in early adulthood on endometrial cancer have not been elucidated. Here, we conducted a case-control study comprising 222 histologically diagnosed incident endometrial cancer cases and 2162 age- and menstrual-status matched non-cancer controls.

**Methods:**

Information on current body weight, weight and height at age 20 years, and lifestyle/environmental factors was obtained from a self-administered questionnaire. Subjects were classified into 3 groups according to change in body mass index (BMI, kg/m^2^) from age 20 years to enrollment (≤0 [reference], 0–3, and >3 kg/m^2^). The effects of adult BMI change and obesity in early adulthood were evaluated using an unconditional logistic regression model adjusted for potential confounders.

**Results:**

A high BMI at age 20 (BMI ≥25, BMI <25 as reference) was significantly positively associated with endometrial cancer risk (*P* = 0.005), as was a BMI increase during adulthood (0–3 BMI change, multivariate odds ratio [OR] = 1.28, 95% confidence interval [CI] = 0.88–1.87; >3 BMI change, OR = 2.02, 95% CI = 1.38–2.96; *P*-trend < 0.001). Parity and BMI at age 20 appeared to modify the effect of weight gain on cancer risk, albeit without statistical significance. This positive association of weight gain with risk was observed only for endometrioid adenocarcinoma.

**Conclusions:**

The results show that endometrial cancer is positively associated with obesity at age 20 and weight gain during adulthood among Japanese women.

## INTRODUCTION

Endometrial cancer is an increasingly common gynecologic cancer in Japanese women; the age-standardized incidence rate increased from 1.4 to 7.3 between 1975 and 2005.^1^ Interestingly, a marked difference in age-specific incidence has been observed among women older than 50 years.^1^ Obesity is an important established risk factor for endometrial cancer,^2^^,^^3^ with many studies showing that current obesity is associated with increased risk for endometrial cancer due to the effect of adiposity on the synthesis and bioavailability of endogenous sex steroid hormones, mainly estrogens.^4^^–^^8^

Although the overall prevalence of overweight (body mass index [BMI] ≥25 [kg/m^2^]) in Japanese women has not changed during the last 20 years, there has been a heterogeneous trend in BMI change across generations: BMI in Japanese women older than 50 years has increased, whereas mean BMI in women aged 20 to 39 has decreased.^9^^,^^10^ These trends in age-specific incidence and age-specific BMI suggest a potential association between weight gain during adulthood and endometrial cancer. Although several epidemiologic studies have reported a positive association with weight gain,^5^^,^^8^^,^^11^^–^^16^ the interaction between weight gain during adulthood and various potential confounders, such as parity and physical activity, has remained unclear.

Here, we conducted a hospital-based, case-control study to examine the association of endometrial cancer risk with BMI at age 20 and weight gain from age 20 years. We also investigated whether these associations differed with regard to possible confounding factors.

## METHODS

### Subjects

The cases comprised 222 patients who had received a new histologic diagnosis of endometrial carcinoma between January 2001 and November 2005 at Aichi Cancer Center Hospital (ACCH) in Japan. With regard to histologic subtype, 177 cases were endometrioid adenocarcinoma (79.7%), 31 were other adenocarcinomas (14.0%), and 14 were unknown (6.3%). Mixed epithelial and mesenchymal tumors were excluded due to insufficient information on tumor etiology. Controls (*n* = 2162) were randomly selected and matched by age (±4 years) and menstrual status (premenopause, perimenopause, or postmenopause) to cases at a 1:10 case-control ratio from 11 814 women who were free of cancer (58 cases were matched with 9 instead of 10 controls each). All subjects were enrolled using the framework of the Hospital-based Epidemiologic Research Program at Aichi Cancer Center (HERPACC), as described elsewhere.^17^^–^^19^ Briefly, using a self-administered questionnaire we collected information on lifestyle factors from all first-visit outpatients aged 20 to 79 years at ACCH who were recruited for HERPACC between January 2001 and November 2005. Patients were also asked about their lifestyle when healthy or before their current symptoms developed. We did not enroll apparently ill patients who had subjective difficulty in answering the questionnaire. A trained interviewer checked responses. Approximately 90% of eligible subjects responded to the questionnaire. Outpatients were also asked to provide blood samples. Our previous study indicated that the lifestyle patterns of first-visit outpatients conformed with those of a randomly selected sample of the general population of Nagoya City.^20^ The data were loaded into the HERPACC database and routinely linked with the hospital-based cancer registry system to update data on cancer incidence. We obtained informed consent from all participants, and the Institutional Ethical Committee of Aichi Cancer Center approved this study.

### Assessment of anthropometric factors and other exposure data

The HERPACC questionnaire includes anthropometric factors as well as other lifestyle factors, body weight when healthy or before current symptoms developed, height, and body weight at age 20. In the questionnaire, respondents were asked: “current (pre-illness) weight?” and “weight at around age 20?” BMI at enrollment was calculated as weight in kilograms divided by the square of height in meters (kg/m^2^) from self-reported height and weight. Change in body weight and BMI from age 20 years to enrollment was calculated as: (current body weight) − (body weight at age 20) and (current BMI) − (BMI at age 20), respectively. Weight change per year from age 20 to enrollment was calculated as [(current body weight in kg) − (body weight at age 20 in kg)]/(age at enrollment − 20). This variable was designed to investigate the association between velocity of weight change and endometrial cancer risk.

In our previous study, we assessed the validity of self-reported values versus measured values for 100 patients randomly selected from among 173 patients with thyroid cancer by comparing current height, weight, and BMI on the HERPACC questionnaire with the respective values measured and recorded in the medical record by hospital staff on admission to ACCH. Self-recorded and measured values for current height, weight, and BMI were highly correlated, with Pearson’s correlation coefficients of 0.978, 0.910, and 0.913 for women, respectively.^21^

Smoking and drinking habits were categorized into 3 categories: never, former, and current. Former smokers and drinkers were defined as ever-smokers and ever-drinkers and included with current smokers and drinkers, respectively, in the analysis. Menstrual status was classified as premenopausal, perimenopausal, or postmenopausal, and premenopausal and perimenopausal women were included in the premenopausal group for the analysis. Postmenopause was defined as the absence of a menstrual cycle for at least 1 year.

### Statistical analysis

To evaluate the strength of associations between body weight change and risk of endometrial cancer, odds ratios (ORs) with 95% confidence intervals (CIs) were estimated using unconditional logistic models adjusted for potential confounders. To improve statistical efficiency in stratified analysis, we used unconditional logistic models after confirming the consistency of results from the conditional and unconditional models. For subgroup analysis, subjects were classified by BMI change into 3 groups (≤0, 0–3, and >3 kg/m^2^). Change in body weight per year from age 20 to enrollment was also divided into 3 groups (≤0, 0–0.19, 0.19–3.50 kg/year, based on median values among controls who had experienced weight gain during adulthood). The potential confounders that were adjusted for in the multivariate analyses were age, smoking habit (never or ever), drinking habit (never or ever), regular exercise (yes or no), age at menarche (≤12, 13–14, or ≥15 years), duration of menstruation (years, tertile), parity (0, 1–2, ≥3), and a history of diabetes (yes or no), contraceptive use (yes or no), and hormone replacement therapy (yes or no). Current BMI and BMI age at 20 were classified into 2 groups (<25 or ≥25 kg/m^2^) based on our previous study.^15^ We defined overweight as a BMI of 25 kg/m^2^ or higher. Missing values for any covariate were treated as dummy variables in the logistic model. Differences in categorized demographic variables between cases and controls were assessed by the chi-square test. Age, age at menarche, current BMI, BMI at age 20, duration of menstruation, and parity were compared in cases and controls by the Mann-Whitney test. To estimate risks for subgroups, we conducted analysis stratified by menstrual status, regular exercise, parity (0 or ≥1), BMI at age 20 (<25 or ≥25 kg/m^2^), and histologic subtype (endometrioid or other carcinoma). A *P* value less than 0.05 was considered to indicate statistical significance. We used STATA version 10.1 (Stata Corp., College Station, TX, USA) for all analyses.

## RESULTS

The baseline characteristics of the 222 endometrial cancer patients and 2162 controls are shown in Table 1. Median age was 56.0 years for both cases and controls. Smoking status did not differ between groups: the prevalence of ever-smokers among cases and controls was 14.9% and 18.9%, respectively. A drinking habit was significantly less common among cases (*P* = 0.002). Current BMI and BMI at age 20 were higher among cases than among controls (*P* < 0.001). Regarding reproductive factors, low parity was more prevalent (*P* < 0.001) and duration of menstruation was longer among cases than among controls (*P* < 0.001). A history of diabetes was more common in cases. There was no difference between groups in regular exercise, age at menarche, history of hypertension, history of oral contraceptive use, or history of hormone replacement therapy use.

**Table 1.** Characteristics of subjects

Characteristic	Cases	(%)	Controls	(%)	*P* value
Number	222		2162		

Histology					
Endometrioid (%)	177	(79.7)			
Other carcinoma (%)	31	(14.0)			
Unknown (%)	14	(6.3)			
Age, years					0.771
(median [min–max])	56.0 (25–79)		56.0 (20–79)		
<40 (%)	37	(16.8)	297	(13.7)	0.306
40–54 (%)	56	(25.2)	631	(29.2)	
≥55 (%)	129	(58.1)	1234	(58.1)	
Smoking status					
Ever (%)	33	(14.9)	1751	(18.9)	0.153
Never (%)	187	(84.2)	409	(81.0)	
Unknown (%)	2	(0.9)	2	(0.1)	
Drinking status					
Ever (%)	61	(27.5)	1331	(38.4)	0.002
Never (%)	159	(71.6)	830	(61.6)	
Unknown (%)	2	(0.9)	1	(0.1)	
Current body mass index					<0.001
(median [min–max])	23.1 (13.4–40.9)		21.6 (13.3–40.9)		
<25 kg/m^2^ (%)	152	(68.5)	1804	(83.4)	<0.001
≥25 kg/m^2^ (%)	65	(29.3)	342	(15.8)	
Unknown (%)	5	(2.3)	16	(0.7)	
Body mass index at age 20					0.001
(median [min–max])	20.8 (14.8–34.3)		20.3 (14.9–34.2)		
<25 kg/m^2^ (%)	196	(88.3)	2043	(94.5)	0.001
≥25 kg/m^2^ (%)	17	(7.7)	70	(3.2)	
Unknown (%)	9	(4.1)	49	(2.3)	
Regular exercise					
No (%)	69	(31.1)	618	(28.6)	0.440
Yes (%)	150	(67.6)	1512	(69.9)	
Unknown (%)	3	(1.4)	32	(1.5)	
Menstrual status					
Premenopausal (%)	78	(35.2)	738	(34.1)	0.709
Postmenopausal (%)	141	(63.5)	1410	(65.2)	
Unknown (%)	3	(1.4)	14	(0.7)	
Age at menarche, years					0.241
(median [min–max])	13.0 (9–19)		14.0 (9–22)		
≤12 (%)	62	(27.9)	555	(25.7)	0.340
13–14 (%)	106	(47.8)	997	(46.1)	
≥15 (%)	47	(21.2)	556	(25.7)	
Unknown (%)	7	(3.2)	54	(2.5)	
Duration of menstruation, years					<0.001
(median [min–max])	36.0 (11–47)		35.0 (6–50)		
≤32 (%)	57	(25.7)	732	(33.9)	0.001
33–37 (%)	69	(31.1)	780	(36.1)	
>37 (%)	83	(37.4)	574	(26.6)	
Unknown (%)	13	(5.9)	76	(3.5)	
Parity					<0.001
(median [min–max])	2 (0–4)		2 (0–7)		
0 (%)	63	(28.4)	347	(16.1)	<0.001
1–2 (%)	123	(55.4)	1318	(61.0)	
≥3 (%)	32	(14.4)	488	(22.6)	
Unknown (%)	4	(1.8)	9	(0.4)	
Diabetes history					
No (%)	207	(93.2)	2080	(96.2)	0.033
Yes (%)	15	(6.8)	82	(3.8)	
Hypertension history					
No (%)	186	(83.8)	1889	(87.4)	0.129
Yes (%)	36	(16.2)	273	(12.6)	

History of contraceptive use					
No (%)	208	(93.7)	2011	(93.0)	0.295
Yes (%)	8	(3.6)	114	(5.3)	
Unknown (%)	6	(2.7)	37	(1.7)	
History of hormone replacement therapy					
No (%)	198	(89.2)	1963	(90.8)	0.401
Yes (%)	20	(9.0)	161	(7.5)	
Unknown (%)	4	(1.8)	38	(1.8)	

Table 2 shows the association of endometrial cancer risk with current BMI and BMI at age 20. A higher current BMI and a higher BMI at age 20 were associated with increased risk: as compared with women with a BMI less than 25, the multivariate OR of women with a current BMI of 25 or greater was 2.22 (95% CI = 1.59–3.09, *P* < 0.001), while that of women with a BMI of 25 or greater at age 20 was 2.30 (1.29–4.11, *P* = 0.005).

**Table 2.** Impact of current BMI and BMI at age 20 years on endometrial cancer risk

Category	Case/control	Age-adjusted OR (95% CI)	Multivariate OR (95% CI)^a^
Current BMI (kg/m^2^)			
<25 kg/m^2^	152/1804	1.00 (Reference)	1.00 (Reference)
≥25 kg/m^2^	65/342	2.26 (1.65–3.10)	2.22 (1.59–3.09)
Unknown	5/16		
*P* for trend		<0.001	<0.001

BMI at age 20 years (kg/m^2^)			
<25 kg/m^2^	196/2043	1.00 (Reference)	1.00 (Reference)
≥25 kg/m^2^	17/70	2.53 (1.46–4.40)	2.30 (1.29–4.11)
Unknown	9/49		
*P* for trend		0.001	0.005

^a^Multivariate models adjusted for age, smoking, drinking, regular exercise, age at menarche, duration of menstruation, parity, diabetes history, history of oral contraceptive use, and history of hormone replacement therapy.

Table 3 shows the associations of endometrial cancer risk with BMI and body weight change from age 20 to study enrollment. Regarding BMI change, women who had a BMI increase of 0 to 3 kg/m^2^ or greater than 3 kg/m^2^ had a higher risk of endometrial cancer than did those with no BMI increase, with ORs of 1.28 (95% CI = 0.88–1.87) and 2.02 (1.38–2.96), respectively (*P*-trend < 0.001). A significant positive association with greater body weight change per year was consistently observed from age 20 to enrollment (*P*-trend = 0.001) and was not changed by adjustment for BMI or body weight at age 20 (*P*-trend < 0.001). However, after adjusting for current BMI, these positive associations were attenuated.

**Table 3.** Odds ratios and 95% CI for endometrial cancer stratified according to BMI change and body weight change from age 20 to enrollment

Category	Case/control	Age-adjusted OR (95% CI)	Multivariate OR (95% CI) in model 1	Multivariate OR (95% CI) in model 2	Multivariate OR (95% CI) in model 3	Multivariate OR (95% CI) in model 4
BMI change from age 20 to enrollment (kg/m^2^)						
≤0	57/719	1.00 (Reference)	1.00 (Reference)	1.00 (Reference)	1.00 (Reference)	1.00 (Reference)
0–3	73/804	1.15 (0.80–1.65)	1.28 (0.88–1.87)^a^	1.42 (0.97–2.10)^b^	1.51 (1.02–2.23)^c^	1.26 (0.86–1.84)^d^
>3	82/581	1.79 (1.25–2.56)	2.02 (1.38–2.96)^a^	2.22 (1.50–3.28)^b^	2.43 (1.64–3.60)^c^	1.48 (0.95–2.29)^d^
Unknown	10/58					
*P* for trend		0.001	<0.001	<0.001^b^	0.001^c^	0.075^d^

Body weight change per year from age 20 to enrollment (kg/year)						
≤0	59/720	1.00 (Reference)	1.00 (Reference)	1.00 (Reference)	1.00 (Reference)	1.00 (Reference)
0–0.19	63/685	1.10 (0.75–1.60)	1.18 (0.80–1.76)^a^	1.35 (0.90–2.02)^b^	1.41 (0.94–2.12)^c^	1.20 (0.81–1.79)^d^
0.19–3.50	93/701	1.62 (1.15–2.29)	1.84 (1.28–2.64)^a^	2.07 (1.42–3.00)^b^	2.20 (1.51–3.20)^c^	1.44 (0.96–2.15)^d^
Unknown	7/56					
*P* for trend		0.005	0.001	<0.001	<0.001^c^	0.075^d^

^a^Multivariate models adjusted for age, smoking, drinking, regular exercise, age at menarche, duration of menstruation, parity, diabetes history, history of oral contraceptive use, and history of hormone replacement therapy.

^b^Multivariate models adjusted for age, smoking, drinking, regular exercise, BMI at age 20, age at menarche, duration of menstruation, parity, diabetes history, history of oral contraceptive use, and history of hormone replacement therapy.

^c^Multivariate models adjusted for age, smoking, drinking, regular exercise, body weight at age 20, age at menarche, duration of menstruation, parity, diabetes history, history of oral contraceptive use, and history of hormone replacement therapy.

^d^Multivariate models adjusted for age, smoking, drinking, regular exercise, current BMI, age at menarche, duration of menstruation, parity, diabetes history, history of oral contraceptive use, and history of hormone replacement therapy.

Table 4 shows the joint effect of BMI change on analysis stratified by potential confounders. The positive association between BMI and endometrial cancer risk remained generally consistent after stratification by menstrual status, regular exercise, parity, and BMI at age 20. The multivariate OR of nulliparous women with a BMI change of 0 to 3 kg/m^2^ was 1.80 (95% CI = 0.91–3.57); among those with a change greater than 3 kg/m^2^, it was 3.75 (1.67–8.37). However, the *P* value for interaction was not significant (interaction *P* = 0.090). With regard to BMI at age 20, women with a BMI of 25 or greater at age 20 had higher ORs (0–3 kg/m^2^ BMI change: multivariate OR = 2.50, 95% CI = 0.19–33.10; >3 kg/m^2^ BMI change: 8.02, 0.95–67.51) than did those with a BMI less than 25 (0–3 kg/m^2^ BMI change: 1.29, 0.86–1.91; >3 kg/m^2^ BMI change: 1.97, 1.31–2.95). However, the interactions between BMI change and BMI at age 20 were not statistically significant (interaction *P* = 0.216). On analysis by histologic subtype, positive associations were observed only among patients with endometrioid carcinoma (0–3 kg/m^2^ BMI change: OR = 1.46, 95% CI = 0.95–2.25; >3 kg/m^2^ BMI change: 2.50, 1.62–3.85).

**Table 4.** Effect of BMI change according to age, menstrual status, exercise, parity, BMI at age 20, and histology on endometrial cancer risk

Category	BMI change from age 20 to enrollment (kg/m^2^)			*P* for trend	

	≤0	0–3	>3		
Total (case/control)^a^	57/719	73/804	82/581		
Age-adjusted OR (95% CI)	1.00 (Reference)	1.15 (0.80–1.65)	1.79 (1.25–2.56)	0.001	
Multivariate OR (95% CI)^b^	1.00 (Reference)	1.28 (0.88–1.87)	2.02 (1.38–2.96)	<0.001	

Menstrual status					interaction *P* = 0.334
Premenopausal (case/control)	20/274	35/283	21/166		
Multivariate OR (95% CI)^b^	1.00 (Reference)	2.10 (1.11–3.98)	2.16 (1.02–4.55)	0.036	
Postmenopausal (case/control)	35/437	38/518	60/412		
Multivariate OR (95% CI)^b^	1.00 (Reference)	0.89 (0.54–1.46)	1.82 (1.15–2.88)	0.006	
Unknown (case/control)	2/8	0/3	1/3		
		interaction *P* = 0.910	interaction *P* = 0.383		

Regular exercise					interaction *P* = 0875
No (case/control)	17/215	26/217	23/167		
Multivariate OR (95% CI)^b^	1.00 (Reference)	1.55 (0.79–3.05)	1.83 (0.90–3.72)	0.097	
Yes (case/control)	40/494	46/578	57/404		
Multivariate OR (95% CI)^b^	1.00 (Reference)	1.16 (0.73–1.85)	2.08 (1.31–3.31)	0.002	
Unknown (case/control)	0/10	1/9	2/10		
		interaction *P* = 0.376	interaction *P* = 0.953		

Parity					interaction *P* = 0.090
0 (case/control)	19/177	24/113	18/48		
Multivariate OR (95% CI)^b^	1.00 (Reference)	1.80 (0.91–3.57)	3.75 (1.67–8.37)	0.001	
≥1 (case/control)	37/537	49/690	61/532		
Multivariate OR (95% CI)^b^	1.00 (Reference)	1.01 (0.64–1.60)	1.49 (0.96–2.32)	0.058	
Unknown (case/control)	1/5	0/1	3/1		
		interaction *P* = 0.129	interaction *P* = 0.077		

BMI at age 20					interaction *P* = 0.216
<25 (case/control)	49/663	70/799	77/573		
Multivariate OR (95% CI)^b^	1.00 (Reference)	1.29 (0.86–1.91)	1.97 (1.31–2.95)	0.001	
≥25 (case/control)	8/56	3/5	5/8		
Multivariate OR (95% CI)^b^	1.00 (Reference)	2.50 (0.19–33.10)	8.02 (0.95–67.51)	0.056	
		interaction *P* = 0.164	interaction *P* = 0.263		

Histology					Not assessed
Endometrioid (case/control)	42/719	59/804	69/581		
Multivariate OR (95% CI)^b^	1.00 (Reference)	1.46 (0.95–2.25)	2.50 (1.62–3.85)	<0.001	
Other carcinoma (case/control)	13/719	9/804	7/581		
Multivariate OR (95% CI)^b^	1.00 (Reference)	0.50 (0.20–1.26)	0.54 (0.20–1.44)	0.179	
Unknown (case/control)	2/719	5/804	6/581		

^a^10 cases and 58 controls were excluded from the analyses due to lack of information on BMI.

^b^Multivariate models adjusted for age, smoking, drinking, regular exercise, age at menarche, duration of menstruation, parity, diabetes history, history of oral contraceptive use, and history of hormone replacement therapy.

Table 5 shows the combined effect of BMI at age 20 and current BMI on endometrial cancer risk. As shown in Table 4, obesity at both age 20 and study enrollment increased endometrial cancer risk (OR = 3.45, 95% CI = 1.72–6.92). However, the interaction between BMI at age 20 and BMI at study enrollment was not significant (interaction *P* = 0.704).

**Table 5.** Combined effect of BMI at age 20 and current BMI on endometrial cancer

Category		Case/control	Age-adjusted OR (95% CI)	Multivariate OR (95% CI)^a,b^
BMI at age 20 (kg/m^2^)	BMI at enrollment (kg/m^2^)			
<25	<25	145/1738	1.00 (Reference)	1.00 (Reference)
≥25	<25	3/33	1.10 (0.33–3.67)	1.25 (0.37–4.34)
<25	≥25	51/297	2.07 (1.46–2.92)	2.07 (1.44–2.97)
≥25	≥25	13/36	4.33 (2.25–8.35)	3.45 (1.72–6.92)
	*P* for trend		<0.001	<0.001
	Interaction *P*		0.369	0.704

^a^10 case and 58 controls were excluded from the analyses due to lack of information on BMI.

^b^Multivariate models adjusted for age, smoking, drinking, regular exercise, age at menarche, duration of menstruation, parity, diabetes history, history of oral contraceptive use, and history of hormone replacement therapy.

## DISCUSSION

We found that both BMI at age 20 and current BMI were associated with a significantly increased risk of endometrial cancer. In addition, weight gain from age 20 to study enrollment was associated with a positive risk of endometrial cancer. Further, nulliparous women, or women who were obese at age 20, who had increased body weight during adulthood were likely to have higher ORs, although the interactions between weight gain and these variables were not statistically significant. These positive associations were observed only among cases of endometrioid cancer.

Table 2 shows that current obesity (BMI ≥25) and obesity at age 20 were associated with an increased risk of endometrial cancer. This finding for current obesity is consistent with those of other studies.^2^^,^^5^^,^^15^ In premenopausal women, obesity causes insulin resistance, which has important effects on ovarian androgen synthesis, anovulation, and chronic progesterone deficiency.^2^^,^^7^ Postmenopausal obese women are thought to have higher levels of estrogens derived from extraglandular conversion of androgens.^2^^,^^7^ Furthermore, obese women are more likely to have lower levels of sex hormone-binding globulin (SHBG).^2^^,^^3^^,^^22^ As a consequence, increased levels of bioavailable estrogen might induce development of endometrial cancer among currently obese women. In contrast, premenopausal obese women are likely to have anovulatory cycles and decreased levels of progesterone. In particular, obese women are exposed to prolonged unopposed estrogens during early adulthood and, as a result, they might have an increased risk of endometrial cancer. The mechanism underlying the association of BMI during early adulthood with subsequent endometrial cancer is not well understood. Obesity at an early age might be determined by genetic constitution and energy intake during puberty.^23^^,^^24^ Findings for this association in previous studies have been inconsistent, possibly due to the attenuation of effect by overadjustment for recent body weight.^8^^,^^12^^,^^14^^,^^15^^,^^25^ A better understanding of the effect of body weight at early age requires future study.

Table 3 shows a positive association between BMI increase and weight gain per year. Studies of the associations of these variables with endometrial cancer have been limited. Park et al showed that women with a BMI gain of 35% or greater had a relative risk of 4.12 (95% CI = 2.69–6.30) as compared with a reference group (−5% ≤ BMI change < +5%).^11^ In our previous study, which was independent of the present study, women with a BMI change of 2.50 or greater from age 20 to enrollment had an OR of 1.70 (95% CI = 1.11–2.61) as compared with the reference group (0 < BMI change < 2.50).^15^ Other studies have also shown a positive association between adult weight gain and endometrial cancer, with 2- to 3-fold increased risk.^5^^,^^8^^,^^12^^–^^14^ Although the etiologic role of weight gain during adulthood is not clear, our study confirms these previous results.

We explored the possibility of interaction by selected factors on the association between BMI change and risk of endometrial cancer (Table 4). Although we observed no statistically significant interaction, several factors were suggestive in terms of mechanism. First, nulliparous women who had weight gain during adulthood were likely to have higher ORs as compared with multiparous women. Second, the impact of BMI change was greater in obese women at age 20 than in nonobese women at that age. In addition, this association was evident only in cases of endometrioid cancer. These findings may be of value in future epidemiologic and biologic studies.

There are several potential limitations in our study that should be considered. First, we used self-reported height and body weight. Previous validity studies of body size among Japanese women have reported that women tend to overestimate height and underestimate weight.^26^ However, correlation coefficients in our validity study were high and considered acceptable.^21^ Second, self-reported body weight at age 20 may be inaccurate. However, the fact that obesity at age 20 is not generally regarded as a risk factor for endometrial cancer likely precludes the possibility of recall bias regarding body size. Third, case-control studies are subject to information bias, although the HERPACC system is less vulnerable to this bias than are typical hospital-based studies, because data for most or all patients are collected before diagnosis. Fourth, because the study was conducted under a hospital-based case-control design, the possibility of inadequate comparability between cases and controls depended on whether the control population was the source population from which the cases arose. However, we selected cases and controls from the same hospital, and almost all these patients live in the Tokai area of central Japan. Validity in the HERPACC study was confirmed in our previous study.^20^ Finally, our study had a modest sample size; thus, replication of our findings in other studies is required.

In conclusion, this case-control study suggests that weight gain in adulthood increases the risk of endometrial cancer among Japanese. Further, a similar association was observed after stratification by potential confounders. This higher risk of endometrial cancer with increased adult weight might be exacerbated in women who were already obese at age 20, although this effect was not statistically significant in the present research. To prevent endometrial cancer, our findings support the importance of weight control starting in early adulthood. Further investigation of these findings is warranted.
